# 54443 Pharmacogenomic Profiling of East and West African Populations

**DOI:** 10.1017/cts.2021.663

**Published:** 2021-03-30

**Authors:** Linsey Jackson, Ruben Bonilla Guerrero, Arjun Athreya, Lewis R. Roberts

**Affiliations:** 1 Mayo Clinic; 2 Admera Health

## Abstract

ABSTRACT IMPACT: Genomic variation likely plays a role in differences in rates of adverse reactions and efficacy for African populations, and this research will add to the understudied field of pharmacogenomics in African populations. OBJECTIVES/GOALS: We aim to characterize the frequency of variants in clinically relevant genes in East and West African populations to assess the prevalence of potential drug-gene interactions. METHODS/STUDY POPULATION: Our pilot study will consist of 100 Somali patients enrolled at Mayo Clinic Rochester and 100 Ghanaian patients recruited at Teaching Hospitals in Ghana. Germline DNA will be extracted from pre-existing blood samples. Sequencing will be performed using Admera Health’s PGxOne Plus test, interrogating a panel of 62 genes. Variants will be reported along with the predicted response for a list of drugs. Differences between frequencies of variants in East and West African populations will be analyzed. We will look for correlations with reported adverse reaction rates. We will then compare our findings with allele frequency data from publicly available data bases. Additionally, we will analyze the flanking regions of the panel for novel variants, using machine learning to predict gene-drug interactions. RESULTS/ANTICIPATED RESULTS: African populations are known to have more genetic diversity than any other population. Additionally, only African-Americans, African-Caribbeans from Barbados, Esan and Yoruba Nigerians, Gambians, Kenyans, and Sierra Leoneans are accounted for within the publicly available data bases most often used for variant studies. Thus, it is anticipated that we will find differences in the variant allele frequencies of our populations compared to European allele frequencies, and differences in frequencies between the East and West African populations. In the 200 base pair flanking regions that are sequenced in the assay along with the known variant regions, we may find novel previously unreported variants. DISCUSSION/SIGNIFICANCE OF FINDINGS: The lack of knowledge of pharmacogenomic variation in African populations contributes to ethnic disparities in patient outcomes. This study addresses this gap by adding to our comprehension of variants in clinically relevant genes, giving insight into underlying mechanisms of ethnicity-based drug responses.

